# Optimization of enzyme-assisted extraction of anthocyanins from eggplant (*Solanum melongena L.*) peel

**DOI:** 10.1016/j.fochx.2023.100643

**Published:** 2023-03-14

**Authors:** PR. Amulya, Rayees ul Islam

**Affiliations:** Department of Post Harvest Engineering and Technology, Faculty of Agricultural Sciences, Aligarh Muslim University, Aligarh, India

**Keywords:** Enzyme- assisted extraction, Eggplant peel, Cellulase, Total anthocyanin content, Total phenolic content, Antioxidant activity

## Abstract

Enzyme-assisted extraction (EAE) of total yield (TY), total anthocyanin content (TA), antioxidant activity (AOA) and total phenolic content (TPC) from eggplant peel was optimized using response surface methodology. Enzyme concentration (5–15 %), extraction temperature (35–60 °C) and maceration time (1–4.5 hrs) were used to optimize the process of extraction using a Central-Composite design. Optimum values of TY (71.45 %), TAC (578.66 mg C3G L^-1^), TPC (2040.87 mg GAE L^-1^), DPPH (79.92 %) and FRAP (29.90 mmol AAE/100 g) were obtained for the optimized extraction parameters viz., temperature (37.32 °C), enzyme concentration (5%) and extraction time (1 h). Further, a comparative study was also done between conventional extraction and enzyme-assisted extraction of eggplant peel. It was observed that the responses of the extract obtained by conventional method showed significant variation from that obtained by EAE indicating the superiority of latter.

## Highlights

• Enzyme-assisted extraction (EAE) was used to extract anthocyanins from eggplant peel.
• EAE depicted the highest total yields and anthocyanin content than conventional solvent extraction.
• EAE of eggplant extract possessed substantial phenolic content and antioxidant capacity.
• EAE can provide an alternate to the conventional extraction method.

## 1 Introduction

Currently, the food industry produces a significant quantity of waste and by-products that can’t be consumed directly. These waste and by-products come from a variety of sources and include various plant parts, such as peel, stem, leaf, seed, kernel, etc.(Estrella-Osuna, Tapia-Hernández, Ruíz-Cruz, Márquez-Ríos, & de Ornelas-Paz, 2022). The eggplant (*Solanum melongena L.)* is a widely cultivated vegetable that is appreciated for its unique flavor, texture, and nutritional properties. About 50 million metric tonnes of eggplants are grown on >1,800,000 ha of land around the world (Gürbüz, Uluişik, Frary, Frary, & Doğanlar, 2018). However, the processing of eggplant generates a considerable amount of waste, including its peel, which is often discarded as a byproduct. Eggplant peel represents a significant source of bioactive compounds such as polyphenols, flavonoids, and dietary fibres, which possess antioxidant, antimicrobial, and anticancer properties. In recent years, several studies have investigated the potential use of eggplant peel extracts or powders in food, pharmaceutical, and cosmetic industries (Karimi, Kazemi, Samani, & Simal-Gandara, 2021).

Anthocyanins are a group of water-soluble pigments responsible for the purple color of eggplant skin. These pigments are widely distributed in nature and are known for their antioxidant and anti-inflammatory properties (Ma, Du, Li, Yang, & Zhu, 2021). Eggplant contains several types of anthocyanins, including delphinidin, petunidin, and malvidin. These compounds are concentrated in the skin of the eggplant, and their levels increase as the fruit ripens (Yarmohammadi, Rahbardar, & Hosseinzadeh, 2021). In addition to their role in coloration, anthocyanins in eggplant have been associated with a range of potential health benefits. Studies suggest that these compounds may help to protect against chronic diseases such as cardiovascular disease, cancer, and diabetes by reducing oxidative stress and inflammation in the body (Salehi et al., 2020). The extraction of anthocyanins from eggplant can be challenging, as these compounds are located in the cell vacuoles and are tightly bound to cell wall components (Kumar, Dahuja, Sachdev, Tomar, Lorenzo, Dhumal, & Radha, 2022). Traditional methods of extraction, such as solvent extraction, may lead to low yields and loss of quality. Therefore, alternative methods are needed to efficiently extract anthocyanins from eggplant. In this research article, we investigated the potential of enzyme-assisted extraction to improve the extraction efficiency of anthocyanins from eggplant.

Enzyme-assisted extraction involves the use of enzymes to break down the cell wall components and release the target compounds from the plant cells. Enzymes used in this process include cellulases, hemicellulases, xylanases, proteases, α–amylases, β–glucosidases and pectinases (González, Carrera, Barbero, & Palma, 2022). Enzyme-assisted extraction has been shown to improve the yield and quality of anthocyanins extracted from plant materials, including fruits and vegetables. Compared to traditional methods of extraction, such as solvent extraction, enzyme-assisted extraction has several advantages, including reduced extraction time, lower solvent consumption, and improved quality of the extracted compounds (Domínguez-Rodríguez, Marina, & Plaza, 2021). Additionally, enzyme-assisted extraction is a more environmentally friendly method of extraction, as it generates less waste and uses milder extraction conditions (Kleekayai et al., 2023). Enzyme-assisted extraction of anthocyanins have been reported in various plant sources (Xu et al., 2016; Alavarsa-Cascales et al., 2022, González et al., 2022). The effectiveness of enzyme-assisted extraction depends on various factors, such as the type and concentration of the enzyme used, extraction temperature, and pH (Nadar, Rao, & Rathod, 2018). Optimal conditions for enzyme-assisted extraction should be determined for each specific plant material to obtain maximum yield and quality of anthocyanins.

In this context, the present study aimed to optimize the conditions of enzyme-assisted extraction, such as enzyme concentration, extraction time and temperature, to maximize the yield and quality of anthocyanins extracted from eggplant. Our findings may provide a new approach for the extraction of anthocyanins from eggplant and contribute to the development of functional food ingredients with potential health benefits.

## 2 Materials and methods

### 2.1 Material collection and storage

Matured and uniformly coloured eggplants were purchased from a native market of Aligarh, India. Foreign matter, dirt, soil particles etc, present in eggplants were removed by washing in running water and were then dried using a clean towel. The eggplants were then peeled manually with the help of a knife and dried in a tray drier at 45 ^0^C. The dried eggplant peels were ground in a laboratory grinder, sieved through a 0.5 mm mesh and were stored in an airtight container at −18 °C until further use. The cellulase enzyme used in the EAE of eggplant peel was provided in powered form by Biolaxi Corporation, Mumbai, India having activities of the order of 1,00,000 CMC (carboxymethylcellulose) unit per g (CMCU/g). Analytical research grade chemicals and reagents (ethanol, citric acid monohydrate, potassium chloride, sodium acetate, hydrochloric acid, gallic acid, Folin Ciocalteau’s reagent, sodium carbonate, methanol, FRAP reagent and DPPH (2, 2- diphenyl-1-picrylhydraxyl) were used in the experiments.

### 2.2 Extraction experiments

#### 2.2.1 Conventional solvent extraction (CSE)

Extraction of anthocyanins was accomplished adopting the method of Hosseini, Gharachorloo, Ghiassi-Tarzi, and Ghavami (2016) with slight modifications. Briefly, 3 g sample was extracted with a solvent mixture of water, ethanol and citric acid in the ratio 50:48:2 (v/v). A constant solid solvent ratio of 1:20 was used throughout the experiment. The extraction of eggplant peel was done in a shaker water bath incubator (Yorco Sales Pvt. Ltd. India, Model: YSI–417). The crude extract obtained by filtering the mixture through Whatman’s filter paper No. 1 was centrifuged in a refrigerated centrifuge (Remi CPR-24 Plus) at 7000 rpm at 5 ^0^C for 15 min. The extract was then refrigerated for 1 day to precipitate large molecules if any and then again centrifuged to obtain a pure extract. The solvent was then removed from the extract and was further concentrated to constant volume at 35 °C in the rotary vacuum evaporator (Kshitij R&D Lab Instruments, India) to prevent degradation of anthocyanin pigments. The concentrated crude extract of eggplant peel was kept in a freezer below 4 ^0^C till analysis.

#### 2.2.2 Enzyme-assisted extraction (EAE) and its experimental design

Enzyme-assisted extraction of eggplant peel was done to improve the anthocyanin recovery using cellulase enzyme. For EAE, central composite design was used to study the effect of temperature (T), enzyme concentration (EC), and extraction time (t). The levels of different variables are given in Table 1. The present experimental design comprised of 20 combinations using 6 central points to assess their effect on four responses namely, total extract yield (TY), total anthocyanins content (TAC), total phenolic content (TPC) and in vitro antioxidant activity (AOA), DPPH & FRAP. Enzyme cellulase was added to samples followed by solvent extraction to enhance the extraction process. The data was fitted to the second order regression equation as given:

**Table 1:** Levels of coded and uncoded factors for the experimental design.

Variables	Factors	−1	+1	0	-α	+α
Temperature, T (°C)	A	35	60	47.5	35	60
Enzyme concentration, EC (% E/S)	B	5	15	10	5	15
Time, t (hrs)	C	1	4.5	2.75	1	4.5

## y = β_0_ + $\sum_{i=1}^{k}\beta_{i}x_{i}$ + $\sum_{i=1}^{k}\beta_{\mathrm{ii}}x_{\mathrm{ii}}^{2}$ + $\sum_{i<j}\sum \beta_{\mathrm{ij}}x_{i}x_{j}$ + ε

where, y is the predicted response, β_0_ is the intercept, β_i_, β_ii_ and β_ij_ are linear, quadratic and interaction coefficients, respectively, and x_i_ and x_j_ are the coded independent variables.

### 2.3 Analytical methods

#### 2.3.1 Total yield (TY)

Total yield (TY) of the extract was determined by weighing the final dried crude extract and was expressed in percent (%) using the formula given below. The results were conducted in triplicates.

$$\mathrm{TYin}\%db=\frac{\mathrm{Weightofextract}}{\mathrm{Sampleweight}}\times 100$$

#### 2.3.2 Quantification of total monomeric anthocyanin content (TAC)

Total anthocyanins content of the extract was determined by pH-differential method following the protocol of Chen et al. (2020) with slight modifications. Briefly, samples were mixed in potassium chloride (0.10 M) buffer of pH 1.0 and sodium acetate (0.5 M) buffer of pH 4.5. These two solutions were allowed to equilibrate for 15 min at room temperature. The absorbance of the solutions was measured at 520 nm and 700 nm, respectively, in a UV–Vis-spectrophotometer (UV–Vis spectrophotometer, Model: UV5704SS) against distilled water (blank). Cyanidin 3- glucoside was used as a standard and the results were expressed as milligrams Cyanidin 3- glucoside equivalents/litre. TAC was calculated using the following formula.

$$\mathrm{Monomericanthocyanincontent}\left(mg/litre\right)=\frac{A\times MW\times DF}{\left(\varepsilon \times l\right)}\times 1000$$

Where,

A = (A_520_ – A_700_) _pH 1.0_ – (A_520_ – A_700_) _pH 4.5_.

MW is the molecular weight.

DF is the dilution factor.

ε is the molar absorptivity.

l is the path length (1 cm).

#### 2.3.3 Determination of total phenolic content (TPC)

The total phenolic content (TPC) of the eggplant extracts was determined by Folin-Ciocalteu spectrophotometrically method following the procedure of Okmen et al. (2009). Breifly, 2 ml of sample was added to 10 ml of 2 N (10%) Folin-Ciocalteau’s reagent and allowed to equilibrate for 3 min in dark. This was followed by the addition of 8 ml sodium carbonate (0.7 M) and incubated for 2 h at room temperature in the dark. The absorbance of the mixture was measured at 765 nm in UV–VIS spectrophotometer (UV–Vis spectrophotometer, Model: UV5704SS). The results of each sample were conducted in triplicates. Total phenolic content of the extracts were expressed in mg gallic acid equivalents (GAE)/litre.

#### 2.3.4 Determination of antioxidant activity (AOA) by DPPH free radicals scavenging activity

*In-vitro* antioxidant activity of the extract was determined using DPPH (2, 2-diphenyl-1-picrylhydraxyl) assay adopting the method of Macedo et al. (2021). Absolute methanol was used as a reference. An aliquot of the extract was diluted at 4 mg/mL in methanol and 1 ml of the diluted extract was then treated with 2 ml of 0.2 mM methanolic solution of DPPH. The reaction mixture was shaken vigorously and was allowed to equilibrate in the dark for 30 min at room temperature. The decrease in absorbance of the mixture was recorded at 517 nm in UV–VIS spectrophotometer until a plateau of the reaction was reached.

#### 2.3.5 Determination of antioxidant activity (AOA) by ferric reducing antioxidant power (FRAP)

The ferric reducing antioxidant power assay was determined following the method of Swer, Mukhim, Bashir, and Chauhan (2018). Briefly, diluted sample extract (0.1 ml) was mixed FRAP reagent (3 ml). The mixture was mixed well and allowed to equilibrate for 4 min in dark. The absorbance was then recorded at 594 nm in UV–VIS spectrophotometer against blank (FRAP). Results were calculated as mmol ascorbic acid equivalent per 100 g dry weight (mmol AAE/100 g DW).

### 2.4 Statistical analysis

For designing the experiment and subsequent statistical analysis Design-Expert software 11.0 trial version (Stat-Ease Inc., Minneapolis, MN, USA) was used. The statistical significance of the models, lack of fit and regression for optimization of extraction conditions were evaluated using analysis of variance (ANOVA). All experiments were done in triplicates.

## 3 Results and discussions

### 3.1 Effect of enzyme-assisted extraction parameters on TY

**Fig. 1:**
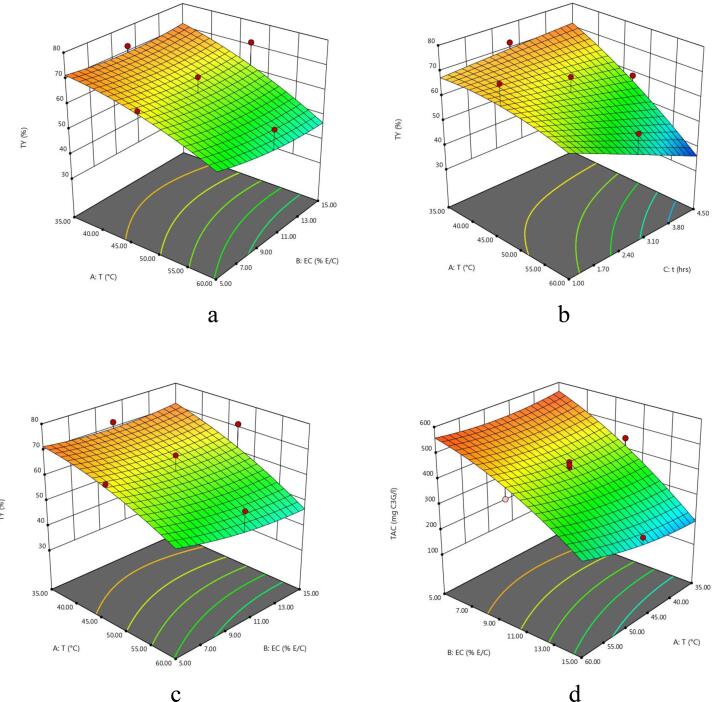

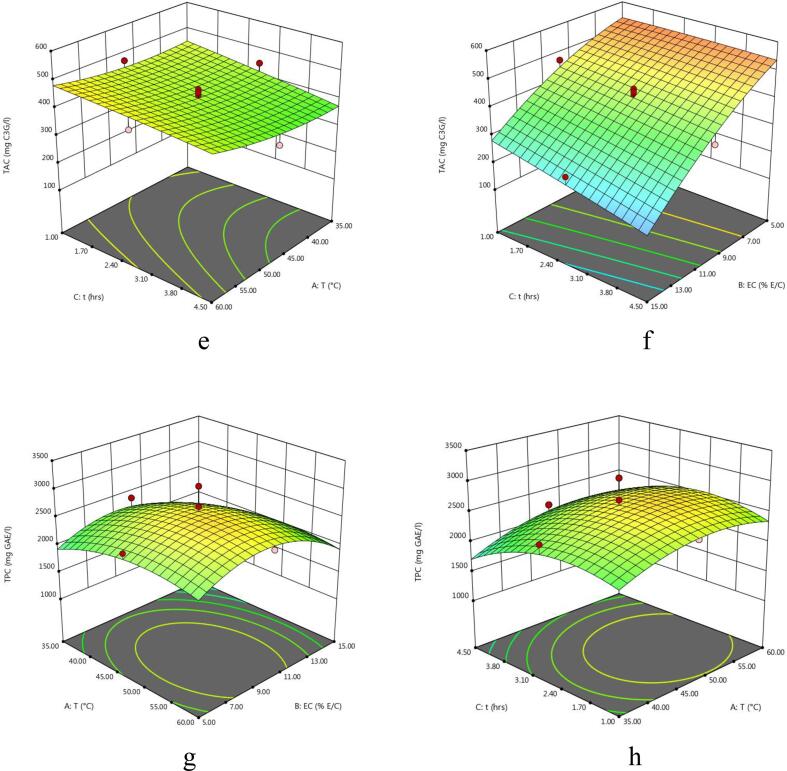

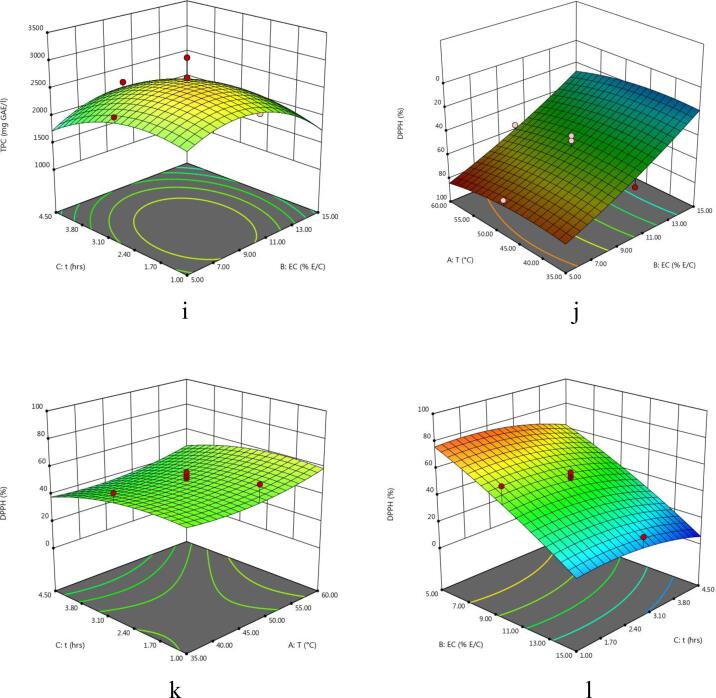

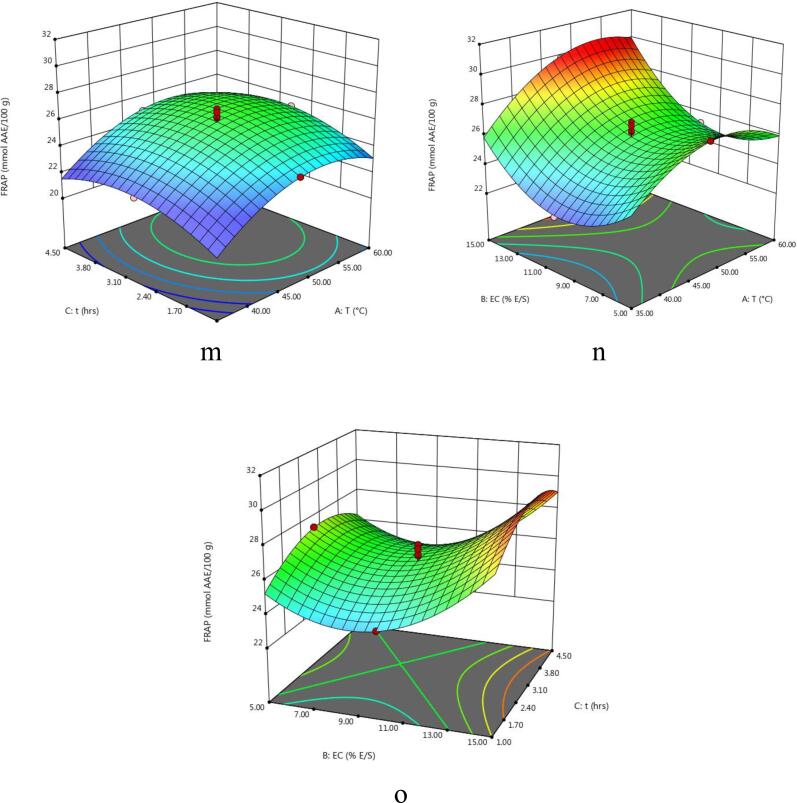
(a-o) 3D Response surface plots for the effects of temperature (A), enzyme concentration (B), and time (C) on total yield (TY), total monomeric anthocyanin content (TAC), total phenolic content (TPC) and antioxidant activity (AOA) (FRAP and DPPH).

The experimental values of TY of the extract obtained from enzyme-assisted extraction of eggplant peel at various experimental conditions varied between 34.17 and 75.5% (Table 2). The regression model of coded variables depicting the effect of extraction parameters on the TY is given in Table 3. Also, the F-value, p-value, determination of coefficient (R^2^), significant values, regression coefficients etc are shown in Table 3, Table 4. It can be observed that extraction variables showed significant linear effects for A and C and interactive effects of extraction variables were found to be significant for AC and all quadratic effects were non-significant for TY from eggplant. The effect of temperature and enzyme concentration, temperature and time and extraction time and enzyme concentration on total yield of eggplant peel extract is shown in 3D plots (Fig. 1a – c). Total yield of the extract decreased significantly with increase in extraction temperature. However, the total yield of the extract increased slightly with increase in enzyme concentration and extraction time. Similar results were reported by Swer et al. (2018) in nepalensis L. fruit. Also, the results reported by Puri, Sharma, Barrow, and Tiwary (2012) in EAE of steviosides from *Stevia rebaudiana* leaves are in agreement with our data. The authors were of the opinion that the negative effect of extraction temperature and time on total yield might result from denaturation of enzymes at higher temperatures.

**Table 2:** Central Composite Design and experimental data of TY, TAC, TPC, AOA using EAE.

Run	Extraction conditions			Experimental results				
	A (°C)	B (% E/S)	C (h)	TY (%)	TAC (mg C3G/l)	TPC (mg GAE/l)	DPPH (%)	FRAP (mmol AAE/100 g
1	60	5	1	68.17	566.08	2080	83.07	23.87
2	35	5	1	70.50	559.41	1960	75.60	22.51
3	47.50	10	1	75.50	470.91	2460	63.79	23.89
4	35	15	1	65.50	242.98	1310	12.95	23.98
5	60	15	1	53.67	332.72	1740	24.37	27.76
6	47.50	5	2.75	67.00	499.71	2400	70.91	27.31
7	47.50	10	2.75	58.34	451.69	2410	53.88	26.23
8	60	10	2.75	57.17	421.23	2460	55.55	25.26
9	47.50	10	2.75	56.83	400.69	2700	41.20	26.31
10	35	10	2.75	72.17	445.86	2380	57.95	22.38
11	47.50	10	2.75	68.15	428.75	3060	51.93	26.89
12	47.50	10	2.75	56.84	412.07	2520	45.34	26.64
13	47.50	10	2.75	58.33	440.85	2430	56.33	23.89
14	47.50	10	2.75	52.67	408.88	2410	45.75	25.70
15	47.50	15	2.75	71.34	256.81	1970	26.09	29.81
16	35	5	4.50	73.00	564.01	1190	65.95	23.65
17	60	5	4.50	39.67	585.30	1710	77.75	25.10
18	47.50	10	4.50	58.84	349.43	2250	28.45	24.88
19	35	15	4.50	74.34	114.30	1180	5.90	24.89
20	60	15	4.50	34.17	267.15	1580	17.15	29.28

A: Temperature; B: Enzyme concentration; C: Time; TY: Total yield; TAC: Total Anthocyanins Content; TPC: Total Phenolic Content; AOA: Antioxidant activity; EAE: Enzyme-assisted extraction; C3G: Cyanidin-3-glucoside; GAE: Gallic acid equivalent; FRAP: Ferric reducing antioxidant property; AAE: Ascorbic acid equivalent.

**Table 3:** Analysis of variance (ANOVA) for the overall effect of independent variables on each response.

Source	DF	TY		TAC		TPC		DPPH		FRAP	
		p- value	F- value	p- value	F- value	p- value	F- value	p- value	F- value	p- value	F- value
Model	1	0.0140^**^	4.49	0.0001*	26.42	0.0002*	12.45	0.0001*	14.72	0.0002*	13.57
A	1	0.0008*	22.42	0.0452^**^	5.24	0.0374^**^	5.75	0.1604 ^ns^	2.30	0.0002*	32.15
B	1	0.3939 ^ns^	0.79	0.0001*	210.81	0.0364^**^	5.83	0.0001*	121.06	0.0003*	29.52
C	1	0.0337^**^	6.05	0.0217^**^	7.38	0.0295^**^	6.44	0.0327^**^	6.14	0.0394^**^	5.61
AB	1	0.4193 ^ns^	0.71	0.0496^**^	4.98	0.7491 ^ns^	0.11	0.8873 ^ns^	0.021	0.0342^**^	6.01
AC	1	0.0121^**^	9.36	0.4375 ^ns^	0.65	0.5364 ^ns^	0.41	0.8620 ^ns^	0.032	0.7554 ^ns^	0.10
BC	1	0.4472 ^ns^	0.63	0.0467^**^	5.15	0.1721 ^ns^	2.16	0.9766 ^ns^	0.090	0.9786 ^ns^	0.0753
A^2^	1	0.5416 ^ns^	0.40	0.0405^**^	1.00	0.0830 ^ns^	3.71	0.3556 ^ns^	0.94	0.0014*	19.00
B^2^	1	0.6578 ^ns^	0.21	0.1206 ^ns^	2.88	0.0033^**^	14.69	0.5048 ^ns^	0.48	0.0002*	33.76
*C*^2^	1	0.9788 ^ns^	0.7433	0.8915 ^ns^	0.020	0.0341^**^	6.02	0.2689 ns	1.37	0.0104^**^	9.90

A: Temperature; B: Enzyme concentration; C: Time.

DF: Degree of Freedom.

Level of significance: * Significant at P < 0.01, **Significant at P < 0.05; ^ns^Not significant at P > 0.05.

**Table 4:** Predicted second order polynomial equations of regression models and other statistical terms for responses in eggplant peel extract using EAE.

Response	Second order polynomial equation	R^2^	Adj-R^2^	Pred-R^2^	Adeq. Precision
TY	62.03–10.27A-1.93B-5.33C-2.04AB-7.42AC + 1.92BC	0.7921	0.6962	0.5266	10.827
TAC	419.50 + 24.59A − 156.05B − 29.19C + 26.83AB + 9.72AC − 27.26BC + 20.51A2-34.77B2 − 2.87C2	0.9596	0.9233	0.7205	18.457
TPC	2615.91 + 155.00A − 156.00B − 164.00C + 23.75AB + 46.25AC + 106.25BC − 237.27A2 − 472.27B2 − 302.27C2	0.9180	0.8443	0.7213	10.039
DPPH	50.21 + 3.95A + 28.68B − 6.46C + 0.42AB + 0.52AC + 0.088BC + 4.82A2 − 3.44B2 − 5.82C2	0.9298	0.8666	0.5754	13.413
FRAP	25.91 + 1.39A + 1.33B − 0.58C + 0.67AB + 0.088AC + 0.075BC − 2.03A^2^ + 2.71B^2^ − 1.47C^2^	0.9243	0.8562	0.8889	13.637

### 3.2 Effect of enzyme-assisted extraction parameters on TAC

Total anthocyanins content of the extract was quantified in terms of cyanidin-3-glucoside and expressed in mg C3G/l of extract. The experimental values of TAC of the extract obtained from enzyme-assisted extraction of eggplant peel at various experimental conditions varied between 114.3 and 585.3 mg C3G/l of extract. The minimum TAC (114.3 mg C3G/l) was found at 35 °C, 15% EC and 4.5 h and maximum TAC (585.3 mg C3G/l) was found at 60 °C, 5% EC and 4.5 h (Table 2). Influence of three independent variables on TAC was significant (*p* < 0.0001) and is demonstrated by the second-order polynomial regression equation given in Table 4. Also, the F-value, p-value, determination of coefficient (R^2^), significant values etc. are shown in Table 3, Table 4. It can be observed that extraction variables showed significant linear effects whereas AB and BC displayed significant interactive effects. However, all the quadratic effects except for temperature showed a non-significant effect (p > 0.05).

The effect of interactive extraction factors viz, temperature and enzyme concentration, temperature and time and extraction time and enzyme concentration on TAC of eggplant peel extract is illustrated in 3-D plots (Fig. 1d – f). Overall, temperature played a vital role on the maximum recovery of anthocyanins released form enzyme treated egg plant. Both extraction temperature and time and overall interaction effect depicted a positive effect on the extraction of TAC as can be seen in polynomial equation (Table 4). Tran, Khang, Le, Nguyen, and Bach (2017) also observed similar patterns reporting increase in extraction of anthnocyanins with increase in temperature and time on TAC of extracts obtained by EAE of purple sweet potatoes. On the other hand, enzyme addition increased extraction of anthocyanins initially up to a certain level which was followed with no increase in anthocyanin recovery upon the further increment in the enzyme concentration. According to Lotfi, Kalbasi-Ashtari, Hamedi, and Ghorbani (2015) the intercellular pigments like anthcyanins become more accessible for extraction as a result of the ezymatic breakdown of plant cell wall matrix. A negative quadratic effect of temperature was seen on the extraction of TAC. According to Cacace & Mazza (2003), anthocyanins degraded and yields were reduced as temperature was raised above 30 to 35 °C. The softening of plant tissues is improved with rise in temperature which in turn enhances the rate of mass transfer of pigments thus improving the anthocyanin solubility of eggplant into the extraction solvent. However, more rise in temperature can decrease the anthocyanin yield owing to the thermal destruction of anthocyanin pigments.

### 3.3 Effect of enzyme-assisted extraction parameters on TPC

The efficacy of EAE of the eggplant peel extract was assessed for the recovery of total phenolic content and was expressed in terms of mg gallic acid equivalents (GAE)/litre of extract. The experimental values of TPC of the extract obtained from enzyme-assisted extraction of eggplant peel at various experimental conditions are presented in Table 1. Influence of three independent variables on TPC was significant (*p* < 0.0001) and is demonstrated by the second-order polynomial regression equation given in Table 4. The quadratic regression model of coded variables depicting the effect of extraction parameters on the TPC is given in Table 1. Various statistical terms like F-value, p-value, determination of coefficient (R^2^), significant values, regression coefficients etc are also shown in Table 3, Table 4. It can be observed that extraction variables showed significant (p < 0.05) linear effects for A and B, whereas AB and AC displayed non-significant (p > 0.05) interactive effects.

The effect of independent variables on the total phenolic content of the extract is depicted in Fig. 1**g** – i. Total phenolic content of the extract obtained varied between 1180 and 3060 mg GAE/L of extract. Slight increase in TPC of the eggplant peel extract was noticed with increase in enzyme concentration. Total phenolic content of the extract increased with increase in temperature and macerating time. On the other hand, interactive effects of temperature and enzyme concentration and temperature and time showed a non-significant (p > 0.05) positive effect on the recovery of TPC of eggplant peels. A study conducted by (Kumar et al., 2022) attained highest value of TPC of black carrot by increasing the values of temperature (45–55 °C), time (50–70 min), and enzyme concentration (0.19–0.20%). Furthermore, enzyme-assisted extraction produced higher phenolic compounds from olive pomace (Macedo et al., 2021).

### 3.4 Effect of enzyme-assisted extraction parameters on AOA

The antioxidant activity of the extract obtained from eggplant peel was measured in terms of FRAP (mmol AAE/100 g dw) and % inhibition of DPPH. Also, the effect of temperature, enzyme concentration and maceration time of extraction on the antioxidant activity of the extract was studied. Antioxidant activity of the extract obtained from enzyme-assisted extraction of eggplant peel varied between 5.9 and 83.07 for DPPH and 22.38 and 29.81 for FRAP (Table 2).

The quadratic regression model of coded variables and various statistical terms depicting the effect of extraction parameters on the DPPH radical scavenging assay & FRAP are also shown in Table 3, Table 4. Moreover, Fig. 1j-o, describes the effect of the extraction variables on *in-vitro* antioxidant activity (DPPH &FRAP) of the extracts obtained from eggplant peel. It was observed that enzyme concentration showed a negative effect on FRAP and DPPH radical scavenging potential of eggplant peel extract as described in polynomial equation (Table 4). This increasing trend of radical scavenging potential with an increase in enzyme concentration is in agreement with the study reported by Swer and Chauhan (2019). These researchers examined the effect of cellulase enzyme on the antioxidant activity of *Prunus nepalensis* extracts. The results showed that the enzyme treatment improved the antioxidant activity. Furthermore, extraction temperature displayed positive effect and macerating time showed a negative impact on AOA of eggplant peel extract. The decrease in AO of extract with an increase in extraction time might result from the degradation of natural antioxidants during these long exposures. Similar results were also observed by Samaram et al. (2015) of papya seed oil who observed that papaya seed oil with stronger antioxidant activity was recovered with shorter extraction time (0 to 15.3 min) while the longer exposure of extraction (>25 min) resulted in weaker antioxidant activity.

### 3.5 Optimization of extraction parameters and model validation

The models for total yield, anthocyanin content, phenolic content, and antioxidant activity of the eggplant peel extract were analysed and the optimised conditions were obtained which contributed the maximum contents of the responses. The numerical optimization system was used to optimize the extraction variables by superimposing of contour plots of the responses viz; TY, TAC, TPC and AOA. Three solutions that had desirability of 1 were selected and used for validation of the model. The best combination of extraction parameters (Temperature 37.32 °C, Enzyme concentration 5% and Time 1 h) was achieved to attain maximum response properties. The predicted optimum value of each response was 71.45% for TY, 578.66 (mg C3G/l) for TAC, 2040.87 (mg GAE/l) for TPC, 79.92% for DPPH and 29.90 (mmol AAE/100 g) for FRAP under the optimized conditions (T 6). On the other hand, experimental values obtained for different responses of enzyme-assisted extraction of eggplant peel extract were as follows: TY = 70.70 %, TAC = 570.12 (mg C3G/l), TPC = 2010.06 (mg GAE/l), DPPH = 77.40% and FRAP = 30.33 (mmol AAE/100 g). There was<3% relative error between the predicted values and actual experimental data. Therefore response values observed in experiments are in good agreement with the data predicted by the suggested regression models, showing that the formulated models are suitable for prediction of TY, TAC, TPC and AOA of eggplant peel extract with any combination of three extraction variables. Further for the optimized conditions obtained through the design, conventional extraction method was also carried out to compare TY, TAC, TPC and AOA of the extract with enzyme-assisted extraction. It was observed that the responses of the extract obtained by conventional method showed significant variation from that obtained by EAE indicating the superiority of latter. These results are shown in Table 5.

**Table 5:** Responses obtained for optimized levels of variables for EAE and conventional solvent extraction method.

Responses	EAE		CSE
	Predicted values	Actual values	
TY (%)	71.45	70.70 ± 0.46	65.00 ± 0.32
TAC (mg C3G/g)	578.66	570.12 ± 0.61	448.67 ± 0.77
TPC (mg GAE/L)	2040.87	2010.06 ± 0.89	1860.12 ± 0.49
DPPH (%)	79.92	77.40 ± 1.12	41.10 ± 0.98
FRAP(mmol AAE/100 g)	29.90	30.33 ± 1.21	31.65 ± 1.06

EAE: Enzyme-assisted extraction; CSE: Conventional solvent extraction.

## 4 Conclusion

In this study, extraction conditions were optimized by RSM for the recovery of anthocyanin pigments by EAE from eggplant peel using central composite design. Three significant independent variables (temperature, time and enzyme concentration) were selected, and consequently were optimized by CCD. The optimum condition of EAE parameters (Temperature 37.32 °C, Enzyme concentration 5% and Time 1 h) was achieved to attain maximum response properties. The predicted optimum value of each response was 71.45% for TY, 578.665 (mg C3G/l) for TAC, 2040.87 (mg GAE/L) for TPC, 79.91% for DPPH and 29.90 (mmol AAE/100 g) for FRAP. EAE was clearly recognized as an effective way to extract bioactives from eggplant peel namely, total anthocyanins, phenolics, and antioxidant activity. It was evident form the results that EAE outmatched the overall performance of conventional extraction process. EAE facilitated the rupture of eggplant peel cells which resulted in improved extraction yields and reducing the time of extraction as well. EAE can, therefore, undoubtedly prove to be a successful and promising technology for the cost-efficient and environmentally-friendly extraction of anthocyanins from eggplant peel compared to CSE. Keeping in mind the beneficial effects of anthocyanins on human health and the ever-increasing trend of using natural colour gives way for tremendous prospects for its applications in food and other relevant industries.

## Declaration of Competing Interest

The authors declare that they have no known competing financial interests or personal relationships that could have appeared to influence the work reported in this paper.

## Data Availability

No data was used for the research described in the article.
